# Feasibility of multi-domain data acquisition in patients at risk for hypoxic-ischemic brain injury following cardiac arrest: a prospective cohort study

**DOI:** 10.1016/j.resplu.2026.101478

**Published:** 2026-09-04

**Authors:** Dallas Genereaux, Bobo Tong, Penelope M.A. Brasher, Ryan L. Hoiland, Mypinder S. Sekhon, Tison Schoenthal, Noah D. Silverberg, Nishtha Parag, William Panenka, Rebecca Grey, Philip N. Ainslie, Thalia S. Field, Anish R. Mitra, Sophie Stukas, Cheryl L. Wellington, Katie N. Dainty, Carmen Choi, Christopher B. Fordyce, Farzad Moien-Afshari, Erin Camille A. Caritativo, Manraj K.S. Heran, William R. Henderson, Donald E.G. Griesdale

**Affiliations:** a Department of Anesthesiology, Pharmacology & Therapeutics, University of British Columbia, Canada; b Centre for Clinical Epidemiology & Evaluation, Vancouver Coastal Health Research Institute, Canada; c Department of Cellular & Physiological Sciences, University of British Columbia Okanagan, Canada; d Department of Medicine, Division of Critical Care Medicine, University of British Columbia, Canada; e Department of Psychology, University of British Columbia, Canada; f Department of Psychiatry, University of British Columbia, Canada; g School of Health and Exercise Sciences, University of British Columbia Okanagan, Canada; h Department of Medicine, Division of Neurology, University of British Columbia, Canada; i Department of Pathology and Laboratory Medicine, Djavad Mowafaghian Centre for Brain Health, University of British Columbia, Canada; j Department of Pathology and Laboratory Medicine, University of British Columbia, Canada; k Dalla Lana School of Public Health, Institute of Health Policy, Management, and Evaluation, Canada; l Urban patient and family partner, Canada; m Department of Medicine, Division of Cardiology, and the Centre for Cardiovascular Innovation, Vancouver General Hospital, University of British Columbia, Canada; n Epilepsy Clinic, Vancouver General Hospital, Vancouver Coastal Health, Canada; o Department of Radiology, University of British Columbia, Canada

**Keywords:** Hypoxic-ischemic brain injury, Feasibility study, Cardiac arrest, Prognostication

## Abstract

**Introduction:**

Hypoxic-ischemic brain injury is a determinant of neurologic injury and death among survivors of cardiac arrest. While neurophysiologic and biomarker changes may improve prognostication, the feasibility of acquiring multimodal physiologic, biologic, imaging, and patient-centered outcome data across the continuum of care is not established.

**Objective:**

To evaluate the feasibility of (1) patient recruitment; (2) acquiring multi-domain variables; and (3) six-month post-discharge outcomes in a cohort of patients with hypoxic-ischemic brain injury after cardiac arrest.

**Methods:**

We conducted a feasibility study of adults admitted to a critical care unit following cardiac arrest. In-hospital data collection included serum biomarkers, transcranial Doppler, neuroimaging, and electroencephalography. Post-discharge outcomes were assessed in-person or remotely at 6-months, and included outcome assessments, biomarkers, and MRI brain. Feasibility was defined a priori.

**Results:**

Thirty participants were enrolled (June 2022–May 2023). Biomarker samples were obtained in 29/30 (97%) participants on Day 1, 28/29 (97%) evaluable participants on Day 2, 21/25 (84%) on Day 3, and 12/17 (71%) on Day 7. Transcranial Doppler was completed in 23/30 (77%) participants, limited by inadequate acoustic windows and technical failures. All participants underwent either CT or MRI, and 23/30 (77%) had electroencephalography. Modified Rankin Scale was obtained in 27/30 (90%) participants. At least one six-month follow-up assessment was completed in 9/12 (75%) hospital survivors, while the complete in-person multidomain battery was completed in 4/12 (33%). Follow-up MRI was completed in 1/4 in-person follow-up attendees.

**Conclusions:**

Early serum biomarker specimen acquisition was feasible, and remote or proxy-assisted approaches increased six-month outcome ascertainment. However, serial transcranial Doppler, later biomarker sampling, systematic MRI acquisition, and full multidomain follow-up were limited by operational and patient-related barriers, particularly the challenges of in-person assessment.

## Introduction

Approximately 60,000 patients sustain an out-of-hospital cardiac arrest per year in Canada.^1^ Cardiac arrest can result in severe brain injury from absent or decreased cerebral blood flow and oxygen delivery. This is termed hypoxic-ischemic brain injury (HIBI). At six months, over half of those who initially survived their cardiac arrest have either poor neurologic function or have died.^2^ HIBI is the major cause of death in this population.^3^

While survival following cardiac arrest has improved over time, this is primarily attributable to improvements in systems of care^4^: early activation of emergency response, immediate high-quality cardiopulmonary resuscitation,^5^ rapid defibrillation,^6^ and care at a cardiac arrest center.^7^ Several post-resuscitation interventions have also been studied: targeted temperature management,^2^, ^8^ mild hypercapnia,^9^ blood pressure targeting^10^ and oxygen thresholds.^11^ Regrettably, none of these interventions have resulted in improved outcomes. In addition, commonly studied clinical and arrest-related characteristics have not identified subgroups of patients who benefit from specific therapies. It is therefore possible that biologically relevant phenotypes remain unrecognized. Recent approaches, including multimodal biomarker profiling and transcranial Doppler (TCD), may enable more precise patient stratification and reveal subgroups that could benefit from existing or novel therapies.

Furthermore, many studies use neurologic function (e.g., Cerebral Performance Category [CPC] or modified Rankin Scale [mRS]) as primary outcome. While important, these metrics are crude and may overlook subtle but clinically meaningful functional gains. Given the absence of identified subgroups benefiting from existing therapies, and lack of patient-centered outcomes, there is a need for comprehensive studies from reperfusion through post-discharge. Beyond traditional discharge survival, future neuroprognostication should capture complex social, physical, and cognitive aspects of recovery. This aligns with guidelines promoting standardized multimodality prognostication.^12^ Our study therefore assessed feasibility of collecting inpatient physiologic data, TCD, neuroimaging, serial neurologic injury biomarkers, and 6-month outcomes in HIBI patients admitted to critical care following cardiac arrest. This study was not designed to test mechanistic or prognostic associations; rather, it evaluated whether the data streams required for future phenotyping or prognostic studies could be collected across acute hospitalization and post-discharge follow-up.

## Methods

We conducted a prospective cohort study of 30 patients who were admitted to either the intensive care unit (ICU) or cardiac intensive care unit (CICU) and remained unconscious following cardiac arrest. We obtained approval from the University of British Columbia Clinical Research Ethics Board (H22-00208) and registered the study at Open Science Framework (DOI https://doi.org/10.17605/OSF.IO/QBW5K). A protocol-to-report comparison, including planned data domains, implemented procedures, and deviations from the registered protocol, is provided in eTable 1.

### Patient inclusion and recruitment

Adult patients (>16 years) were enrolled following admission to the ICU (40-bed) or CICU (15-bed) at Vancouver General Hospital (a quaternary care referral center) following cardiac arrest due to any cause. Patients were included if they had at least 20 consecutive minutes of spontaneous circulation following resuscitation, and a best Glasgow Coma Score (GCS) equal to or less than 8 after return of spontaneous circulation (ROSC). Patients were excluded if any of the following applied: greater than 72-h following cardiac arrest to time of enrollment, concurrent acute neurologic event (e.g. intracerebral hemorrhage, ischemic stroke, traumatic brain injury, or high cervical spine injury causing arrest), a past history of acquired brain injury (traumatic brain injury, intracerebral hemorrhage, or ischemic stroke), or previous cardiac arrest without full neurologic recovery. We also excluded patients if the decision had been made to withdraw or withhold care. Informed consent was obtained from the participant’s substitute decision maker either at enrollment or through deferred consent. All clinical management, including neuroprognostication, was at the discretion of the attending physician.

### Data collection while in hospital

We collected data on demographics, pre-admission comorbidities, and home medications for each participant. Baseline data also included location of arrest (in-hospital vs out-of-hospital), time from arrest to ROSC, duration of cardiopulmonary resuscitation, shocks delivered, and presumed etiology of arrest. Blood samples for biomarker analyses were collected from an arterial line upon enrolment (Day 1) and at 24-h (Day 2), 48-h (Day 3), and 6-days (Day 7) post enrolment. Blood samples were only collected if the participant was still admitted to the ICU or CICU at the defined timepoint. We originally planned to obtain bilateral TCD (NovaGuide 2® Intelligent Ultrasound, NeuraSignal, CA, USA) measures of middle cerebral artery blood velocity as a proxy for cerebral blood flow on each of the first three admission days. However, owing to initial technical difficulty with the machine, this was revised to at least one TCD during the first three days. Performance of and date of clinical neuroimaging (CT head, MRI brain) and electroencephalography (EEG) obtained as part of usual care in hospital were recorded.

### Six month follow-up assessment

Participants who survived to hospital discharge were invited to participate in a follow-up assessment at the six-month timepoint following their initial cardiac arrest. Study personnel contacted participants via telephone and email to invite them to participate. Participants could complete the follow-up assessments either in-person at the University of British Columbia Brain Research Outcomes Assessment Center in Vancouver BC, or remotely through telephone or Zoom, along with an online questionnaire. Participants were able to have a caregiver assist with completion of some of the assessment forms (assessment via proxy).

Six-month outcome assessments were chosen based on the Core Outcome Set for Cardiac Arrest.^13^ Specific domains and measures are presented in eTable 2.

In-person assessments included performance-based and self-reported outcome assessments, venipuncture blood sample collection for biomarker analysis, and an MRI brain scan for those eligible. The remote assessment included clinical assessments and self-reported outcomes. Patients were reimbursed for local travel expenses and received the following stipends: $100 for the full clinical in-person battery, $50 for MRI, $20 for 6-month bloodwork, and $25 for the abbreviated remote battery.

### Feasibility outcomes

The primary goal of this study was to assess the feasibility of patient recruitment, and obtaining >90% collection of biomarkers, TCD, and obtaining six-month clinical outcomes. Completeness was calculated as the total percentage of successfully collected variables for all evaluable participants. For blood-based biomarkers of neurologic injury, only those patients who were still admitted to the ICU or CICU were considered evaluable. For example, a patient who was deceased or discharged to the ward on day 5 was not included in the total number when considering the total percentage of biomarkers that were collected for day 7 biomarkers. We defined feasibility *a priori* as having successful blood specimen acquisition for neurologic injury biomarkers at >90% of evaluable timepoints during the study period, TCD data for >90% of all evaluable patients, 6-month clinical outcomes for >90% of evaluable participants, 6-month patient reported and performance metrics for >80% of evaluable participants that survived to discharge.

### Biomarker analysis

All blood samples were labeled by study identification and transported directly to the Core Facility for Neurology Biomarker Innovation at the University of British Columbia and stored for batch analyses. Serum levels of glial fibrillary acidic protein (GFAP), a marker of astroglial damage and blood–brain-barrier injury, neurofilament light (NfL) and tau, both markers of axonal injury, and ubiquitin carboxyl terminal hydrolase L1 (UCH-L1), a marker of neuronal cell body injury, were quantified using the Neurology-4-Plex B (N4PB) Advantage Assay (cat ID 103345, lot 504005) using Simoa technology on the HD-X platform from Quanterix Ltd. Assays were performed according to the manufacturer’s instructions; the technician performing the experiments was blinded to participant status.

### Data analysis & sample size

The original protocol specified a convenience sample of 20 participants. During study conduct, recruitment was extended to 30 participants because resources permitted additional enrolment and because early feasibility experience demonstrated substantial downstream attrition, particularly for post-discharge and in-person assessments. The final sample therefore represents the maximum number of participants that could be recruited with available resources and was intended to improve precision around stage-specific feasibility estimates. Participants were listed as shockable rhythm if ventricular fibrillation (VF) or ventricular tachycardia (VT) was listed as the initial rhythm, or if no rhythm noted but a shock was delivered by paramedics. Time to ROSC was calculated as the total of “no flow” (minutes of bystander cardiopulmonary resuscitation [CPR]) and “low flow” time (minutes of CPR by paramedics). All analyses were performed in R v4.3.2 (R Foundation for Statistical Computing, Austria) or STATA 19.5 (StataCorp LLC, College Station, TX).

As a post-hoc exploratory analysis, we compared average biomarker concentrations between patients who survived to hospital discharge and those who did not. Because biomarker availability decreased substantially after Day 2, and to limit survivorship bias, we restricted this comparison to Day 1 and Day 2. Consistent with methodological recommendations for pilot studies we calculated the between-group difference (Alive − Dead) with corresponding 80% confidence intervals to describe the range of plausible effects.^15^ Protocol-specified exploratory analyses by sex and presenting rhythm were not pursued because of the small sample size within these strata.

## Results

**Fig. 1:**
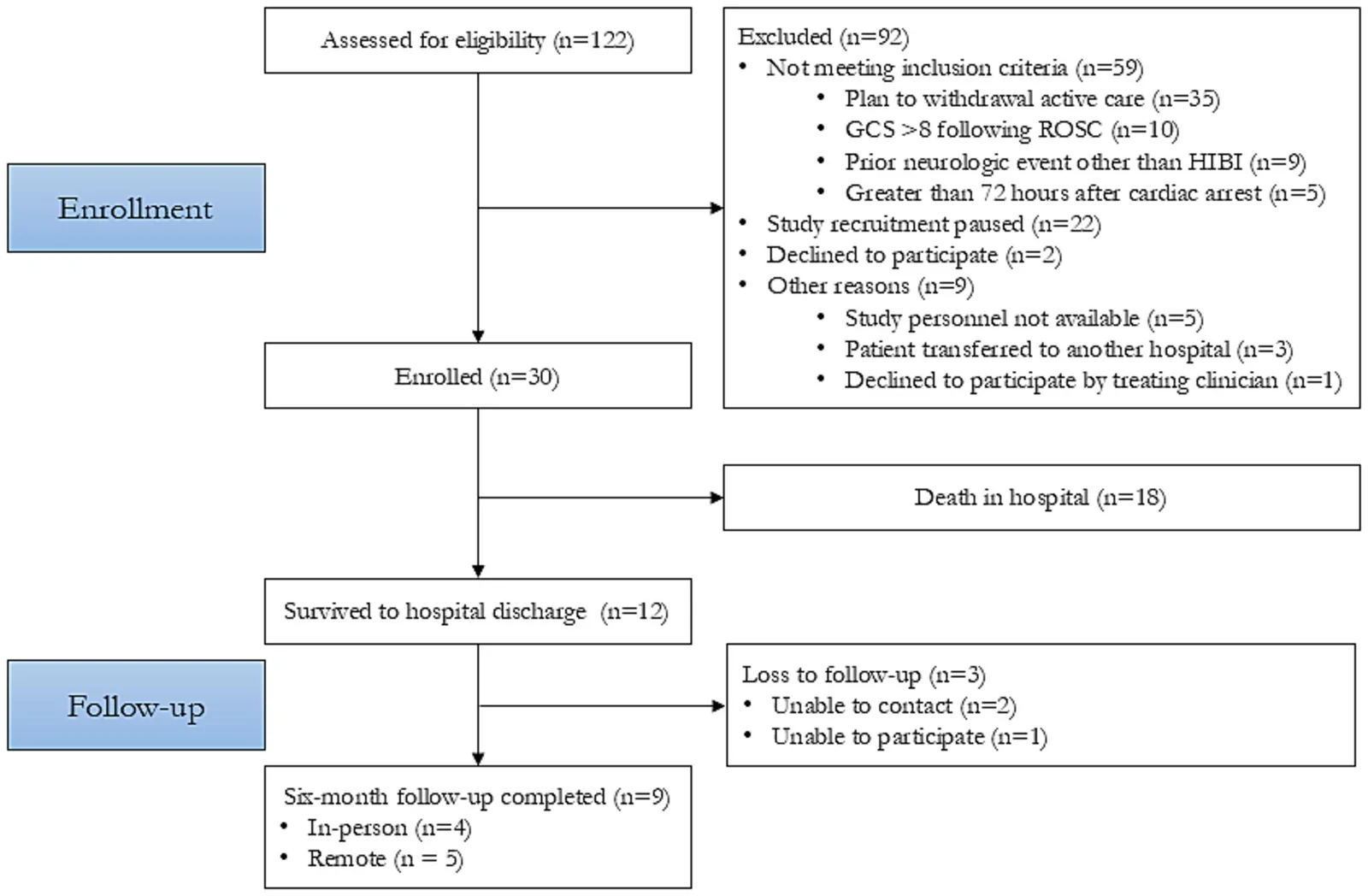
**Overview of recruitment and patient-flow during the study**.

Thirty participants were enrolled from June 2022 to May 2023 (Fig. 1). In total, 122 potential participants were screened for inclusion during the enrollment period. Recruitment was suspended for nearly three months while the TCD machine was undergoing repairs, leading to 22 eligible study candidates being excluded. Excluding this time period, the overall recruitment rate during the study was three participants per month. Participant demographics and clinical characteristics are presented in Table 1. Overall, the cohort had a mean age of 59 years (SD 17) and 7 (23%) were female. The initial rhythm was shockable in 11 (37%). Ten of the patients were enrolled from the CICU.

**Table 1:** Demographics and clinical characteristics of enrolled participants.

	***n* = 30**
Age, mean (SD)	59 (17)
Female sex, *n* (%)	7 (23)
Shockable, *n* (%)	11 (37)
**Etiology of arrest, *n* (%)**	
Myocardial infarction	5 (17)
Hypoxemia	12 (40)
Arrhythmia^a^	6 (20)
Other^b^	7 (23)
APACHE 2 score, median (IQI)	29 (22–34)
**Number of comorbidities**	
0	16 (53)
1	4 (13)
2 or more	10 (33)
Out of hospital location of arrest, *n* (%)	27 (90)
Witnessed arrest, *n* (%)	20 (67)
Time to ROSC in minutes, median (IQI)	18 (10–34)
Initial serum lactate, median mmol/L (IQI)	8.3 (4.5–11.2)
GCS after ROSC, median (IQI)	3 (3–6)
Target temperature day 1, median °C (IQI)	36.7 (36.3–37)
VA-ECMO required, *n* (%)	2 (7)
Active cooling device, *n* (%)	27 (90)
PCI performed, *n* (%)	3 (10)

SD = standard deviation; APACHE = Acute Physiology and Chronic Health Evaluation; IQI = interquartile interval, ROSC = return of spontaneous circulation; GCS = Glasgow Coma Score.

a Arrhythmia = unexplained polymorphic VT, primary arrhythmias, Brugada syndrome, and suspected scar mediated VF/VT.

b Other = includes pulmonary embolism, severe metabolic derangements, presumed cardiogenic (unclear etiology), asthma exacerbation, anaphylaxis, and trauma.

### Feasibility of in-hospital data collection

The completeness of collection for in-hospital variables is presented in eFig. 1. Twenty-three (77%) participants had TCD measurement of middle cerebral artery blood velocity completed at least once during their admission; five patients had TCD monitoring on two days and three had TCD monitoring on three days. This did not meet our pre-specified feasibility threshold of >90%. TCD monitoring was conducted for an average of 38-min [range 3–118, IQI 35–54]. In three patients, we were unable to insonate the middle cerebral artery due to lack of a suitable acoustic window, and we had technical failure of the TCD in 4 patients. This eventually led to a pause in enrollment until the technical issues could be resolved. TCD data are presented in eTable 3. Every participant had an initial CT head completed as part of their clinical care; 16 (53%) participants had at least one follow-up CT scan. Ten (33%) participants had an MRI brain completed during their admission and 23 (77%) had an EEG performed as part of their clinical care. EEGs were requested for the following reasons: possible clinical seizure like event (*n* = 7), evaluation for subclinical seizure (*n* = 8), weaning off sedation (*n* = 4) and to help with neuroprognostication (*n* = 4). EEG interpretation is presented in eTable 4. A denominator-aware summary of feasibility outcomes, including evaluable denominators, completion rates, prespecified feasibility targets, and reasons for incomplete collection, is provided in eTable 5.

### Blood biomarkers of neurologic injury

We obtained biomarkers in 29 of 30 (97%) patients on day-1, 28 of 29 (97%) on day-2, 21 of 25 (84%) on day-3, and 12 of 17 (71%) of patients on day-7. These counts refer to successful blood specimen acquisition at each evaluable timepoint. Analysable biomarker results varied by analyte because of assay-specific availability, and analyte-specific sample sizes are reported in eTable 6. Overall, biomarkers were obtained at 80 of 101 (79%) of all evaluable timepoints which did not meet our prespecified feasibility threshold of >90%. Biomarker levels are presented in eFig. 2 and eTable 6. Mean differences (Alive − Dead) with 80% confidence intervals for biomarker levels on Day 1 and Day 2 are presented in eTable 7. GFAP, UCH-L1, and NfL were higher on average in non-survivors on Day 1 and Day 2, although biomarker distributions overlapped considerably between survivors and non-survivors.

### Feasibility of outcome ascertainment following hospital discharge

Of 30 participants, 15 (50%) were discharged alive from critical care units and 12 (40%) survived to hospital discharge. The median critical care unit and hospital length of stays were 9.5 days [IQI 6.5–12] and 20 days [IQI 11–43], respectively. Overall, mRS scores were obtained in 27 of 30 participants. Of the 12 participants who were discharged alive from hospital, nine agreed and were able to complete the six-month follow-up assessment. Four participants attended an in-person follow-up appointment, while five participants chose to participate via remote assessment. Of the five remote assessments completed, three required a proxy caregiver to help complete questionnaires, one was conducted remotely because the participant was unable to attend in-person appointments, and one was conducted remotely due to participant preference. In the case of proxy-led assessments, measures of depression, anxiety, or PTSD were not administered. The completion rate amongst survivors of the six-month assessments is presented in Table 2. Results are presented in eTables 8–15, and eFigs. 3 and 4.

**Table 2:** Completion of six-month assessment battery in survivors to hospital discharge.

	***n* = 12**
**In-person only assessments, *n***	
NIH toolbox	4
Grooved pegboard	4
CB&M	4
**All assessments, *n***	
TAPS	6
COVID questionnaire	7
CCI	9
mRS	9
WHODAS	9
Self-reported tools	5

Of the four participants who attended the in-person follow-ups, all were able to provide a blood sample and complete the full assessment battery. With regards to follow-up MRI screening, two of the four in-person participants were ineligible for MRI due to implanted devices, and one participant declined follow-up MRI; therefore, follow-up MRI was completed in 1/4 in-person attendees (25%), corresponding to 1/12 hospital survivors (8%). Among the 12 patients who survived to 6-months, 11 were alive at 12-months.

## Discussion

In our prospective cohort study of patients with HIBI following cardiac arrest, we demonstrated near-complete collection of biomarkers at baseline but had attrition at subsequent time-points. While we did obtain TCD in the majority of patients, we did not achieve our *a priori* feasibility target of 90%. Finally, mRS ascertainment was operationally encouraging, although it did not meet the prespecified threshold of >90%, and completion of patient-reported and performance-based outcomes was limited by remote/proxy assessment and low in-person attendance.

For TCD, there were both difficulties with locating adequate acoustic windows in some participants, along with technical issues with the TCD equipment. These difficulties were compounded when trying to monitor multiple participants with overlapping ICU stays, and while this did not lead to missed collection, it does highlight the potential need for multiple devices and operators depending on future study goals. Consequently, we revised the protocol to target a single TCD assessment during ICU admission. The inability to insonate with TCD occurs in between 8% and 20% of patients. Risk factors for the inability to insonate are increased age and female sex.^14^ Thus, the *a priori* feasibility target of 90% may be too high and could be reconsidered in future studies. While we experienced technical failures, this was likely owing to us implementing the novel robotic-assisted TCD. These devices use a signal detection algorithm to help detect and maintain insonation of the middle cerebral artery.^15^ We had no further failures once the technical issues had been fixed by the vendor. Overcoming these obstacles highlights the importance of feasibility work.

Biomarker data collection was largely feasible during the early phase of ICU admission, particularly during the first two days. There was a notable decline in sample acquisition on days 3 and 7; an in-depth review of each missed sample collection was completed; however, it remains unclear what factors may have contributed to this decline. Furthermore, the observed difference between blood specimen acquisition and analyzable biomarker results suggests that future studies should account for both collection feasibility and analyte-specific assay success when estimating sample size and planning serial biomarker analyses.

The contribution of this study is that it evaluates the feasibility of integrating serial biomarker sampling, TCD, usual-care neurodiagnostics, and multidomain six-month follow-up within a single post-cardiac-arrest platform. An encouraging finding was the high completion rate (90%) for overall six-month neurologic outcome which is comparable to, and in some cases exceeds, observational studies in cardiac arrest populations, where follow-up rates have ranged from 42% to 89%.^16^, ^17^, ^18^ While we were able to measure outcomes in multiple domains in 75% of survivors, this was primarily driven through remote and proxy-assisted approaches. Completion of the full multidomain battery, which required in-person assessment, was not feasible. In addition to the measures put-forth in the Core Outcome Set for Cardiac Arrest,^13^ we also assessed the domains of: disability, health-related quality of life, neurobehavioural, anxiety, depression, PTSD, comorbidities, substance use, and somatic symptoms. There are few studies that have attempted to comprehensively measure patient-reported and performance-based outcome measures in this patient population. A study by Amacher and colleagues assessed multiple domains of survivors of cardiac arrest, with a follow-up of 43% at 24-months. Thirty-nine percent of patients in their study had a high-degree of physical impairment as measured by the EQ-5D-5L. Although the absolute number of survivors in whom we could obtain six-month outcomes was small, half were moderately disabled (mRS 3–5). This is also evident as a substantial portion of these assessments required proxy input, and some participants were unable to complete all outcome forms.

The inclusion of both in-person and remote assessments in our feasibility methodology was crucial to maximizing outcome ascertainment, and our experience aligns with published strategies to mitigate loss to follow-up in critically ill cohorts. However, follow-up MRI scans proved infeasible, with only one participant completing the screening. Given the low availability of patients for in-person assessments, and low uptake of MRI, we recommend that future protocols should prioritize remote follow-up assessments to maximize participation. Also, there is certainly selection bias introduced by differences in functional status between participants who were able to attend in-person visits versus those assessed remotely. This discrepancy limits the generalizability of functional outcome data and should be addressed in future study designs. It may be possible in the future to have biomarker bloodwork requisitions sent to participants to be completed at local laboratories, increasing the utility of the six-month biomarker assessment.

This study has several limitations. As a single-center study conducted at a quaternary cardiac arrest center with established research infrastructure, these feasibility estimates may not generalize to centers with different staffing, equipment access, or follow-up resources. While the small sample limits the analysis to primarily descriptive results, the purpose of this study was to assess feasibility and help plan for future research in this area. At the time of protocol development, the degree of stage-specific attrition across data domains was uncertain. A key finding of this study was that evaluable denominators differed substantially across domains because of death, loss of indwelling vascular access (for obtaining biomarkers), inability to attend in-person follow-up, need for proxy-assisted completion, and contraindications to MRI. Furthermore, in-person follow-up participation was low, with only four of 12 hospital survivors attending in person. Among the five remote assessments, one was completed remotely because the participant was unable to attend in person due to geography, and three required proxy caregiver assistance, limiting completion of self-reported and performance-based outcomes. Future studies should use these empiric estimates to prespecify domain-specific denominators, feasibility thresholds, and mitigation strategies.

Based on our findings, ensuring the ability for remote and proxy assessments is key to ensure adequate follow-up. Operational barriers, such as equipment availability and staffing for procedures like TCD and serial biomarker collection, should be proactively addressed through dedicated support, cross-availability of multiple staff, and potentially through additional equipment availability. Obtaining post-discharge MRI is difficult owing to the challenges of in-person assessment, as well as contraindications in this particular patient population (e.g., presence of a pacemaker). If biomarker measurements following discharge are essential, creative strategies, such as local lab requisitions, should be explored to improve collection. Finally, the exploratory findings related to early biomarker trends and clinical outcomes support further investigation of these markers in larger cohorts to assess their potential role in neuroprognostication following cardiac arrest.

## Conclusion

In this single-center feasibility cohort of patients at risk for hypoxic-ischemic brain injury after cardiac arrest, early serum biomarker specimen acquisition was feasible, and remote or proxy-assisted approaches increased six-month outcome ascertainment. However, completion of the full multidomain follow-up battery was not feasible, largely because several components required in-person assessment. Serial transcranial Doppler monitoring, later in-hospital biomarker sampling, systematic inpatient MRI acquisition, and post-discharge MRI were also limited by operational and patient-related barriers. Future studies should use a selective multidomain data-collection strategy, prioritize early in-hospital sampling and remote follow-up, and prospectively plan domain-specific denominators, feasibility thresholds, and mitigation strategies for data streams requiring specialized equipment or in-person attendance.

## Funding

Funding for this project was provided by the 10.13039/100016867ZOLL Foundation. Additional funding was provided from the Jenkins Memorial Award from the Department of Anesthesiology, Pharmacology & Therapeutics at the University of British Columbia.

## CRediT authorship contribution statement

**Dallas Genereaux:** Writing – original draft, Formal analysis. **Bobo Tong:** Writing – review & editing, Formal analysis. **Penelope M.A. Brasher:** Writing – review & editing, Methodology. **Ryan L. Hoiland:** Writing – review & editing, Investigation. **Mypinder S. Sekhon:** Writing – review & editing, Investigation. **Tison Schoenthal:** Writing – review & editing, Investigation. **Noah D. Silverberg:** Writing – review & editing. **Nishtha Parag:** Writing – review & editing, Investigation, Formal analysis. **William Panenka:** Writing – review & editing. **Rebecca Grey:** Writing – review & editing, Project administration, Investigation. **Philip N. Ainslie:** Writing – review & editing. **Thalia S. Field:** Writing – review & editing. **Anish R. Mitra:** Writing – review & editing. **Sophie Stukas:** Writing – review & editing, Investigation. **Cheryl L. Wellington:** Writing – review & editing. **Katie N. Dainty:** Writing – review & editing. **Carmen Choi:** Writing – review & editing. **Christopher B. Fordyce:** Writing – review & editing. **Farzad Moien-Afshari:** Writing – review & editing. **Erin Camille A. Caritativo:** Writing – review & editing, Investigation. **Manraj K.S. Heran:** Writing – review & editing. **William R. Henderson:** Writing – review & editing. **Donald E.G. Griesdale:** Writing – review & editing, Supervision, Methodology, Funding acquisition, Conceptualization.

## Declaration of competing interest

The authors declare the following financial interests/personal relationships which may be considered as potential competing interests: Donald Griesdale reports financial support was provided by ZOLL Foundation. Dallas Genereaux reports financial support was provided by Jenkins Memorial Fund. If there are other authors, they declare that they have no known competing financial interests or personal relationships that could have appeared to influence the work reported in this paper.

## Appendix A Supplementary material

Supplementary Data 1 Additional supplementary feasibility and outcome data.
