# Supplementary material for: Feasibility of multi-domain data acquisition in patients at risk for hypoxic-ischemic brain injury following cardiac arrest: a prospective cohort study
Source: Resusc Plus. 2026 Sep 4;32:101478. doi: 10.1016/j.resplu.2026.101478 (PMC13602316; doi:10.1016/j.resplu.2026.101478)
Supplement: Supplementary file 1 [file mmc1.docx]

**eTable 1:** Protocol-specified data collection and analyses compared with reporting in the current manuscript

**eTable 2:** Six-month follow-up assessments performed

**eTable 3:** Transcranial Doppler values for each patient.

**eTable 4:** Initial electroencephalography (EEG) findings stratified by hospital survival

**eTable 5:** Denominator-aware feasibility summary by data domain and timepoint

**eTable 6:** Biomarker concentrations by time

**eTable 7:** Median [IQI] and mean differences (80% CI) in biomarker concentrations between survivors and non-survivors on Day 1 and Day 2

**eTable 8:** Modified Rankin Scale at 6-months amongst survivors

**eTable 9:** Community Balance and Mobility Scale

**eTable 10:** NIH Toolbox

**eTable 11:** Generalized Anxiety Disorder (GAD - 7), Post-traumatic stress disorder checklist (PCL - 5), Patient Health Questionnaire (PHQ - 15), Somatic Symptom Disorder (SSD - 12) scores amongst survivors.

**eTable 12:** Tobacco, Alcohol, Prescription Medication & other Substance use Tool (TAPS) part 1 amongst survivors who completed the tool (n=6). Answers are individual responses.

**eTable 13:** Tobacco, Alcohol, Prescription Medication & other Substance use Tool (TAPS) part 2 amongst survivors who completed the tool (n=6). Answers are individual responses.

**eTable 14:** Charlson Comorbidity Index amongst 9 respondents

**eTable 15:** COVID Questionnaire

**eFigure 1:** Cleveland dot plot demonstrating the completeness of in-hospital data acquisition

**eFigure 2**: Serum levels of A) GFAP B) tau C) NfL and D) UCH-L1 were quantified on day 1, 2, 3, and 7 post cardiac arrest and at 6-month follow-up in eligible participants. Serial samples from individual patients are linked, with closed squares representing the group median levels per timepoint.

**eFigure 3**: Stacked bar chart of health-related quality of life scores as assessed by EQ-5D-5L

**eFigure 4:** Stacked bar chart of the Patient Health Questionnaire (PHQ - 9)

**eTable 1.** Protocol-specified data collection and analyses compared with reporting in the current manuscript

| **Protocol domain** | **Protocol-specified plan** | **Reported in manuscript** | **Explanation / feasibility interpretation** |
| --- | --- | --- | --- |
| **Sample size** | Convenience sample of 20 patients at Vancouver General Hospital. Protocol rationale focused on excluding an unacceptable monitoring failure rate if no failures were observed. | Thirty participants were enrolled between June 2022 and May 2023. | Modified. Recruitment continued beyond the original convenience sample because resources permitted additional enrolment and to improve precision of feasibility estimates in a population with higher-than anticipated attrition. |
| **Acute serum neurologic injury biomarkers** | Blood samples at enrolment, 24 hours, 48 hours, and 7 days or just prior to line removal. Biomarkers: GFAP, NfL, tau, and UCH-L1. Feasibility target: >90% of eligible timepoints. | Collection was 29/30 on Day 1, 28/29 on Day 2, 21/25 on Day 3, and 12/17 on Day 7; overall 80/101 evaluable timepoints. | Partially achieved. Early serum biomarker collection was feasible, but later sampling declined and overall collection did not meet the >90% feasibility threshold across evaluable timepoints. |
| **PBMC / immune-cell / CyTOF analyses** | Protocol specified peripheral blood mononuclear cell analysis in >90% of eligible timepoints and measurement of immune-cell biomarkers including HLA-DR high monocytes, natural killer T cells, CD4 helper cells, CD8 T cells, and B lymphocytes using CyTOF. | Current manuscript reports serum neurologic injury biomarkers but does not report PBMC/CyTOF immune-cell analyses. | Funding constraints did not permit collection or analysis. |
| **Transcranial Doppler** | TCD following enrolment and at approximately 24 and 48 hours. Feasibility target grouped with CT, MRI, TCD, and EEG in >90% of patients. | Manuscript states bilateral TCD on each of the first three admission days was originally planned but revised to at least one TCD during the first three days because of technical difficulty. TCD was completed at least once in 23/30 participants; five had two days and three had three days. | Modified during study conduct. TCD did not meet the prespecified >90% threshold. Barriers included inadequate acoustic windows, technical failure, equipment repair, and operational burden during overlapping ICU admissions. |
| **Inpatient MRI brain** | Protocol stated that all patients would receive MRI brain at approximately five days after cardiac arrest as part of usual care. | Inpatient MRI brain was completed in 10/30 participants. | Not achieved as written. Systematic inpatient MRI was limited by usual-care decision-making (e.g., clinical stability) and MRI availability / prioritization for clinical care. |
| **Electroencephalography** | EEG was to be obtained in all patients as part of usual care at approximately 24-48 hours after cardiac arrest. | EEG was performed in 23/30 participants and clinical indications for EEG request were listed. | Partially achieved. EEG did not meet the >90% feasibility threshold. Systematic inpatient EEG was limited by usual-care decision-making (e.g., clinical stability) and availability of EEG resources. |
| **Six-month clinical outcomes: survival and mRS** | Protocol specified survival at six months and one year, and neurologic function at hospital discharge and six months measured by modified Rankin Scale. | Manuscript reports hospital discharge survival, critical care discharge survival, and mRS in 27/30 participants. Nine of 12 hospital survivors completed the six-month follow-up assessment. | Operationally encouraging, but did not formally meet the prespecified >90% feasibility threshold. |
| **Six-month patient-reported outcomes** | Protocol specified WHODAS 2.0 and EQ-5D at six months, with in-person assessment preferred and an abbreviated telephone battery available. | Manuscript reports in-person or remote six-month assessments. Five were remote and three required proxy caregiver assistance. Depression, anxiety, and PTSD measures were not administered in proxy-led assessments. | Partially achieved. Remote/proxy assessment supported remote follow-up, but full in-person assessment was not feasible owing to patient morbidity and inability to travel to a central location. |
| **NIH Toolbox / performance-based outcomes** | Protocol specified NIH Toolbox at six months as part of the in-person battery; the abbreviated telephone assessment did not include NIH Toolbox. | Four participants attended in-person follow-up and completed the full assessment battery. | Achieved only among in-person attendees. Not feasible as a universal survivor outcome because most survivors completed remote/proxy follow-up or did not attend in person. |
| **Six-month blood biomarkers** | Protocol specified outpatient six-month blood sampling for participants who elected in-person follow-up, requiring venipuncture. | All four participants who attended in-person follow-up provided a blood sample. | Technically feasible among participants attending in person, but limited by low in-person attendance. Future protocols may require local laboratory collection if six-month biomarkers are important. |
| **Six-month MRI brain** | Protocol specified MRI brain at six-month follow-up for participants presenting for in-person assessment. | Only one follow-up MRI was completed among 12 hospital survivors. Of four in-person attendees, two were ineligible due to implanted devices and one declined. | Not feasible as implemented. Barriers included limited in-person attendance, MRI contraindications, and participant declination. |
| **Exploratory analyses by presenting rhythm and sex** | Protocol specified descriptive analyses for the cohort and by sex and presenting rhythm. Aim 2 planned exploration of shockable vs non-shockable rhythm and sex across TCD, biomarkers, imaging, EEG, and clinical outcomes. | Manuscript reports sex and shockable rhythm descriptively, but exploratory biomarker comparisons are by hospital survival status. | Modified/post hoc. The survival-based biomarker comparison is identified as a post hoc exploratory analysis in the manuscript. Planned sex/rhythm analyses were not pursued because of small sample size within those strata. |
| CT, computed tomography; EEG, electroencephalography; EQ-5D, EuroQol-5D; GFAP, glial fibrillary acidic protein; GCS, Glasgow Coma Scale; ICU, intensive care unit; CCU/CICU, coronary/cardiac intensive care unit; MRI, magnetic resonance imaging; mRS, modified Rankin Scale; NfL, neurofilament light; PBMC, peripheral blood mononuclear cell; TCD, transcranial Doppler; UCH-L1, ubiquitin carboxyl-terminal hydrolase L1; WHODAS, World Health Organization Disability Assessment Schedule. This table focuses on protocol-specified elements that were modified, deferred, incompletely implemented, or specifically raised during peer review. Elements implemented as planned and not central to feasibility interpretation are not exhaustively listed. | | | |

**eTable 2:** Six-month follow-up assessments performed

Performance tests: Grooved pegboard^*^, Community balance & mobility scale^*^

Disability: mRS, WHODAS 2.0

Health-related quality of life: EQ-5D-5L

Neurobehavioural: NIH Toolbox^*^

Anxiety/Depression: PHQ-9, GAD-7

PTSD: PCL-5

Somatic symptoms: PHQ-15, SSD-12

Unclassified: TAPS, CCI, COVID Questionnaire

mRS: modified Rankin Scale Tool. WHODAS 2.0: World Health Organization Disability Assessment Schedule 2.0. TAPS: Tobacco, Alcohol, Prescription Medication, and other Substance use Tool. CCI: Charlson Comorbidity Index. EQ-5D-5L: EuroQol Research Foundation Quality of Life descriptive and visual assessment. PHQ-9: Patient Health Questionnaire for depression. GAD-7: Generalized Anxiety Disorder. PCL-5: Post traumatic stress disorder (PTSD) Checklist. PHQ-15: Patient Health Questionnaire for somatoform disorders. SSD-12: The Somatic Symptom Disorder -B Criteria Scale. Self-reported questionnaires (quality of life, anxiety/depression, PTSD and somatic symptoms) could not be completed by proxy. Asterisk (*) denotes that these tests require in-person assessment.

**eTable 3:** Transcranial Doppler values for each patient.

| Study ID | Day of Study | Minutes of monitoring | FVmean (cm/s) | PI |
| --- | --- | --- | --- | --- |
| 1 | 2 | 55 | 62 | 0.82 |
| 1 | 3 | 41 | 106 | 0.88 |
| 2 | 1 | 13 | 22 | 0.78 |
| 2 | 2 | 10 | 83 | 0.72 |
| 2 | 3 | 7 | 44 | 0.88 |
| 3 | 1 | 69 | 38 | 1.23 |
| 3 | 2 | 3 | 48 | 0.65 |
| 3 | 3 | 12 | 60 | 1.5 |
| 4 | 1 | 95 | 75 | 0.63 |
| 4 | 2 | 29 | 44 | 0.79 |
| 4 | 3 | 35 | 50 | 1.74 |
| 5 | 1 | 37 | 67 | 1.1 |
| 6 | 1 | 19 | 52 | 3.4 |
| 7 | 1 | 44 | 60 | 1.8 |
| 8 | 1 | 60 | 60 | 0.78 |
| 8 | 2 | 39 | 74 | 0.99 |
| 9 | 1 | 118 | 59 | 0.77 |
| 10 | 1 | 33 | 40 | 0.88 |
| 11 | 1 | 12 | 46 | 1.31 |
| 11 | 2 | 28 | 67 | 0.81 |
| 12 | 1 | 36 | 55 | 2.1 |
| 12 | 2 | 21 | 60 | 0.75 |
| 13 | 2 | 55 | 69 | 0.79 |
| 14 | 1 | 28 | 51 | 0.99 |
| 15 | no insonation window | | | |
| 16 | 1 | 77 | 74 | 0.96 |
| 17 | 1 | 31 | 58 | 0.99 |
| 18 | no insonation window | | | |
| 19 | 1 | 36 | 46 | 2 |
| 20 | 1 | 40 | 73 | 0.85 |
| 21 | 1 | 76 | 28 | 1.82 |
| 21 | 2 | 56 | 49 | 2 |
| 22 | 1 | 53 | 49 | 1.3 |
| 23 | 1 | 14 | 34 | 1.1 |
| 24 | TCD not working | | | |
| 25 | TCD not working | | | |
| 26 | 1 | 11 | 32 | 1.44 |
| 27 | 1 | 22 | 85 | 0.98 |
| 28 | TCD not working | | | |
| 29 | TCD not working | | | |
| 30 | no insonation window | | | |
| FVmean = mean flow velocity of the middle cerebral artery, averaged between right and left side; PI = pulsatility index | | | | |

**eTable 4:** Initial electroencephalography findings stratified by hospital survival

| **Finding** | **Alive  (n=12)** | **Died  (n=18)** |
| --- | --- | --- |
| Normal reactive posterior dominant alpha rhythm | 2 |  |
| Poorly regulated and unsustained PDR |  | 2 |
| Absent posterior dominant alpha rhythm | 1 | 9 |
| Reactive to eye opening/closure or stimuli | 1 | 2 |
| Absent to minimal reactivity/variability | 1 | 7 |
| Alpha coma |  |  |
| Generalized suppression/attenuation |  | 6 |
| Burst suppression pattern (off sedation) |  | 1 |
| Burst suppression pattern (on sedation) | 1 | 4 |
| Discontinuous background |  |  |
| Diffuse delta/theta slowing | 6 | 9 |
| Focal delta/theta slowing | 2 | 1 |
| Generalized rhythmic delta activity | 1 |  |
| Generalized periodic discharges | 2 | 5 |
| Lateralized periodic discharges | 2 | 1 |
| Epileptiform discharges | 1 |  |
| Sharp transients | 1 | 1 |
| Triphasic waves | 1 | 1 |
| Electroclinical seizures (e.g. eyelid twitching, tonic eyelid opening, myoclonus) | 2 | 1 |
| Status epilepticus | 1 | 1 |
| Clinical events suspicious for but not definite seizures | 1 |  |
| Clinical events with no EEG correlate |  | 1 |
| EEG = electroencephalography Note that each patient may be represented more than once as they may have multiple findings. | | |

**eTable 5:** Denominator-aware feasibility summary by data domain and timepoint

| **Data domain** | **Timepoint / measure** | **Evaluable denominator** | **Completed, n/N (%)** | **Prespecified feasibility target** | **Target met** | **Main reason for incomplete collection / interpretation** |
| --- | --- | --- | --- | --- | --- | --- |
| Recruitment | Enrolment during active study period | Eligible/screened participants during active recruitment | 30 enrolled; approximately 3/month excluding transcranial Doppler repair pause | No numeric threshold specified | Not prespecified | Outside the equipment-repair pause, recruitment was approximately three participants per month. No numeric recruitment threshold was prespecified. |
| Serum neurologic injury biomarkers | Day 1 | All enrolled participants | 29/30 (97%) | >90% of evaluable timepoints | Yes | One missed baseline sample. |
| Serum neurologic injury biomarkers | Day 2 | Participants still admitted to ICU/CICU and evaluable for sampling | 28/29 (97%) | >90% of evaluable timepoints | Yes | One missed sample. |
| Serum neurologic injury biomarkers | Day 3 | Participants still admitted to ICU/CICU and evaluable for sampling | 21/25 (84%) | >90% of evaluable timepoints | No | Likely limited by loss of indwelling arterial/venous access, ICU/CICU attrition, and operational missed collection; reasons for individual missed samples were not prospectively categorized. |
| Serum neurologic injury biomarkers | Day 7 | Participants still admitted to ICU/CICU and evaluable for sampling | 12/17 (71%) | >90% of evaluable timepoints | No | Likely limited by loss of indwelling arterial/venous access, ICU/CICU attrition, and operational missed collection; reasons for individual missed samples were not prospectively categorized. |
| Serum neurologic injury biomarkers | Overall acute biomarker collection | All evaluable acute biomarker timepoints | 80/101 (79%) | >90% of evaluable timepoints | No | Early collection was feasible, but later timepoint collection declined. |
| Transcranial Doppler | At least one inpatient transcranial Doppler assessment | All enrolled participants | 23/30 (77%) | >90% of patients | No | Limited by inadequate acoustic windows, technical failure, equipment repair, and operational burden during overlapping ICU admissions. |
| Transcranial Doppler | Serial inpatient transcranial Doppler across planned early timepoints | All enrolled participants | 3/30 (10%) completed all three planned days; 8/30 (27%) completed at least two days | Protocol planned enrolment, approximately 24h, and approximately 48h assessments | No | Protocol was modified to target at least one assessment during the first three days because of technical difficulty and operational burden. |
| Neuroimaging | Initial CT head | All enrolled participants | 30/30 (100%) | >90% of patients for CT/MRI/TCD/EEG domain | Yes | Initial CT was obtained as part of usual clinical care. |
| Neuroimaging | Inpatient MRI brain | All enrolled participants | 10/30 (33%) | >90% of patients for CT/MRI/TCD/EEG domain | No | Systematic inpatient MRI was limited by usual-care decision-making, clinical stability, MRI eligibility, and MRI availability/prioritization for clinical care. |
| Electroencephalography | Inpatient EEG | All enrolled participants | 23/30 (77%) | >90% of patients for CT/MRI/TCD/EEG domain | No | Systematic inpatient EEG was limited by usual-care decision-making, clinical stability, and availability of EEG resources. |
| Six-month clinical outcome | Modified Rankin Scale | All enrolled participants | 27/30 (90%) | >90% of evaluable participants | No | Operationally encouraging for global neurologic outcome ascertainment, but did not meet the prespecified >90% threshold. |
| Six-month multidomain follow-up | Any in-person or remote six-month follow-up assessment | Hospital survivors | 9/12 (75%) | >80% of survivors for patient-reported and performance-based metrics | No | Limited by non-participation, morbidity, geography, remote/proxy requirements, and in-person assessment burden. |
| Six-month performance-based outcomes | NIH Toolbox / Grooved Pegboard / CB&M | Hospital survivors | 4/12 (33%) | >80% of survivors if considered part of full performance-based battery | No | Performance-based testing required in-person attendance; most survivors completed remote/proxy follow-up or did not attend in person. |
| Six-month blood biomarkers | Venipuncture blood sample | In-person follow-up attendees | 4/4 (100%) | Not separately specified | Not prespecified | Feasible among participants attending in person; overall survivor-level collection was limited because only 4/12 hospital survivors attended in person. |
| Six-month MRI brain | Follow-up MRI | In-person follow-up attendees | 1/4 (25%) | Not separately specified | Not prespecified | Two in-person attendees were ineligible because of implanted devices and one declined MRI. |
| Twelve-month survival | Survival status at 12 months | Hospital survivors | 12/12 status ascertained; 11/12 alive at 12 months | No numeric threshold specified | Not prespecified | Twelve-month survival was reported as a descriptive clinical outcome rather than a prespecified feasibility threshold. |

**Notes:** Evaluable denominators reflect the population in whom the measure could reasonably be collected at the specified timepoint. For acute serum biomarkers, the denominator was restricted to participants still admitted to ICU/CICU and eligible for sampling through existing indwelling access. Research venipuncture was not part of the acute inpatient collection pathway. Completion refers to blood specimen acquisition; analyzable results varied by biomarker assay and are reported in eTable 6.

For six-month blood biomarkers and MRI brain, the evaluable denominator is restricted to participants who attended in-person follow-up, because these assessments were embedded in the in-person pathway. Only 4 of 12 hospital survivors attended in-person follow-up.

Reasons for incomplete collection were not prospectively categorized for all domains; reason fields summarize known or most plausible feasibility barriers based on study conduct and manuscript reporting.

**Abbreviations:** CB&M, Community Balance and Mobility Scale; CICU, cardiac intensive care unit; CT, computed tomography; EEG, electroencephalography; GFAP, glial fibrillary acidic protein; ICU, intensive care unit; MRI, magnetic resonance imaging; mRS, modified Rankin Scale; NfL, neurofilament light; TCD, transcranial Doppler; UCH-L1, ubiquitin carboxyl-terminal hydrolase L1; WHODAS, World Health Organization Disability Assessment Schedule.

**eTable 6:** Biomarker concentrations by time

| **Biomarker** | **Time** | **n** | **Median [IQI] (pg/mL)** |
| --- | --- | --- | --- |
| GFAP | Day 1 | 28 | 640 [250–1,800] |
| GFAP | Day 2 | 28 | 1200 [350–3,100] |
| GFAP | Day 3 | 21 | 820 [270–2,100] |
| GFAP | Day 7 | 12 | 540 [320–1,200] |
| GFAP | 6-month | 4 | 190 [140–250] |
| tau | Day 1 | 26 | 10 [3–39] |
| tau | Day 2 | 24 | 10 [2–38] |
| tau | Day 3 | 20 | 6 [3–100] |
| tau | Day 7 | 12 | 10 [3–45] |
| tau | 6-month | 4 | 2 [2–3] |
| NfL | Day 1 | 28 | 56 [21–1,300] |
| NfL | Day 2 | 28 | 370 [45–1,800] |
| NfL | Day 3 | 21 | 160 [45–1,400] |
| NfL | Day 7 | 12 | 570 [150–860] |
| NfL | 6-month | 4 | 23 [15–32] |
| UCHL-1 | Day 1 | 26 | 120 [60–330] |
| UCHL-1 | Day 2 | 24 | 88 [29–250] |
| UCHL-1 | Day 3 | 20 | 60 [41–180] |
| UCHL-1 | Day 7 | 12 | 65 [45–160] |
| UCHL-1 | 6-month | 4 | 17 [13–19] |
| IQI = interquartile interval | | | |

**eTable 7:** Median [IQI] and mean differences (Alive – Dead) with corresponding 80% CI on Day 1 and Day 2

| **Biomarker** | **Day** | **Alive  (median [IQI]; n)** | **Dead  (median [IQI]; n)** | **Mean Difference (pg/mL)** | **80% CI (pg/mL)** |
| --- | --- | --- | --- | --- | --- |
| GFAP | 1 | 210 [160–450] n = 12 | 1,530 [650–2,360] n = 16 | −2,700 | −4,300 to −1,100 |
| GFAP | 2 | 420 [190–1100] n = 12 | 1,840 [700–4,180] n = 16 | −72,000 | −163,000 to 19,000 |
| tau | 1 | 5 [2–12] n = 12 | 15 [9–42] n = 14 | −56 | −114 to 3 |
| tau | 2 | 3 [2–14] n = 11 | 33 [8–57] n = 13 | −43 | −87 to 0 |
| NfL | 1 | 18 [13–30] n = 12 | 1,050 [150–1,600] n = 16 | −1,300 | −1,800 to −790 |
| NfL | 2 | 44 [17–114] n = 12 | 1,170 [370–3,850] n = 16 | −2,500 | −3,900 to −1,000 |
| UCHL-1 | 1 | 65 [30–128] n = 12 | 243 [116–517] n = 14 | −164 | −270 to −58 |
| UCHL-1 | 2 | 27 [19–116] n = 11 | 245 [60–307] n = 13 | −182 | −259 to −104 |
| IQI = interquartile interval; CI = confidence interval | | | | | |

**eTable 8:** Modified Rankin Scale at 6-months amongst survivors overall, and by respondent type.

| **Modified Rankin Scale** | **Overall, N = 9*^1^*** | **Participant, N = 6*^1^*** | **SDM/ Other, N = 3*^1^*** |
| --- | --- | --- | --- |
| 0 | 2 | 2 | 0 |
| 1 | 3 | 3 | 0 |
| 2 | 0 | 0 | 0 |
| 3 | 2 | 1 | 1 |
| 4 | 1 | 0 | 1 |
| 5 | 1 | 0 | 1 |

0 = no disability, 1 = no significant disability despite symptoms, 2 = slight disability, unable to carry out all prior activities, 3 = moderate disability requiring some help, but able to walk without assistance, 4 = moderate-severe disability, unable to walk without assistance, 5 = severe disability, bedridden, incontinent and requiring constant nursing care

**eTable 9:** Community Balance and Mobility Scale

| **Community Balance Mobility Scale** | **N = 4***^1^* |
| --- | --- |
| **Unilateral stance- left leg** |  |
| Unable to sustain | 1 |
| ≥ 20.00 sec | 2 |
| 45.00 sec, steady and coordinated | 1 |
| **Unilateral stance- right leg** |  |
| Unable to sustain | 1 |
| 2.00 to 4.49 sec | 1 |
| 45.00 sec, steady and coordinated | 2 |
| **Tandem walking** |  |
| Unable | 1 |
| >3 consecutive steps: heel-toe distance <3” | 1 |
| >3 consecutive steps: in good alignment | 1 |
| 7 consecutive steps: in good alignment | 1 |
| **180 degree tandem pivot** |  |
| Sustains tandem stance but unable to unweight heels or initiate pivot | 1 |
| Initiates pivot but unable to complete 180 degree turn | 1 |
| Completes 180 degree turn but discontinuous pivot | 1 |
| Completes 180 degree turn in a continuous motion and sustains reversed position | 1 |
| **Lateral foot scooting- left leg** |  |
| Unable | 2 |
| 1 lateral pivot | 1 |
| ≥ 3 pivots but < 40 cm | 1 |
| **Lateral foot scooting- right leg** |  |
| Unable | 2 |
| 1 lateral pivot | 1 |
| ≥ 3 pivots but < 40 cm | 1 |
| **Hopping forward- left leg** |  |
| Unable | 1 |
| 1 to 2 hops, uncontrolled | 1 |
| 2 hops, controlled but unable to 1 meter | 1 |
| 1 meter in 2 hops but unable to sustain landing | 1 |
| **Hopping forward- right leg** |  |
| Unable | 1 |
| 1 to 2 hops, uncontrolled | 1 |
| 1 meter in 2 hops but unable to sustain landing | 2 |
| **Crouch & walk** |  |
| Unable to crouch | 1 |
| Descends and rises but hesitates, unable to maintain forward momentum | 2 |
| Crouches and walks in continuous motion, time <=8 sec excess equilibrium reaction | 1 |
| **Lateral Dodging** |  |
| 1 cross-over in both directions in any fashion | 1 |
| 1 or more cycles, but does not contact line every step | 1 |
| 2 cycles, contacts line every step | 1 |
| 2 cycles, contacts line every step 12.00 to 15.00 sec | 1 |
| **Walking & looking- looking left** |  |
| Performs but loses visual fixation at or before 4 meter mark | 1 |
| Performs but loses visual fixation after 4 meter mark | 3 |
| **Walking & looking- looking right** |  |
| Performs but loses visual fixation at or before 4 meter mark | 1 |
| Performs but loses visual fixation after 4 meter mark | 3 |
| **Running with controlled stop** |  |
| Unable to run | 1 |
| Runs, time > 5.00 sec | 1 |
| Runs, time > 3.00 but ≤5.00 sec, unable to control stop | 1 |
| Runs, time > 3.00 but ≤ 5.00 sec, with controlled stop, both feet on line | 1 |
| **Forward to backward walking** |  |
| Performs with reduced speed, time >11.00 sec or requires 4 or more steps to turn | 1 |
| Performs in ≤ 9.00 sec and/or uses protective step during or just after turn | 3 |
| **Walk, look & carry- looking left** |  |
| Performs but loses visual fixation after 4 meter mark | 4 |
| **Walk, look & carry- looking right** |  |
| Performs but loses visual fixation at or before 4 meter mark | 2 |
| Performs but loses visual fixation after 4 meter mark | 2 |
| **Descending stairs** |  |
| Full flight reciprocal, awkward: no railing, no cane | 1 |
| Full flight reciprocal, rhythmical and coordinated: no railing, no cane | 3 |
| **Descending stairs- bonus** |  |
| No bonus | 1 |
| Bonus for carrying basket: no railing | 3 |
| **Step ups x1 step- left leg** |  |
| Steps up and down (1 cycle): unacceptable to look at feet | 1 |
| Completes 5 cycles: unacceptable to look at feet | 1 |
| Completes 5 cycles in >6.00 but <10.00 sec: unacceptable to look at feet | 2 |
| **Step ups x1 step- right leg** |  |
| Step up and down (1 cycle): unacceptable to look at feet | 1 |
| Completes 5 cycles: unacceptable to look at feet | 1 |
| Completes 5 cycles in >6.00 but <10.00 sec: unacceptable to look at feet | 1 |
| Completes 5 cycles in ≤ 6.00 sec, rhythmical: unacceptable to look at feet | 1 |
| **Total score, mean (SD)** | **46 (19)** |
| The Community Balance Mobility Scale ranges between 0 and 96 with higher scores indicating better performance. Healthy young adults will have a score >85 whereas a score < 70 indicates balance-impairment. | |

**eTable 10:** NIH Toolbox (n=4)

| **NIH Toolbox Category** | **Subset** | **Mean (SD)** |
| --- | --- | --- |
| Fluid Composite | Uncorrected standard score | 93 (9) |
|  | Age-corrected standard score | 102 (8) |
|  | Fully- corrected T-score | 50 (6) |
| Crystallized composite score | Uncorrected standard score | 118 (8) |
|  | Age-corrected standard score | 122 (13) |
|  | Fully-corrected T-score | 68 (10) |
| Total composite | Uncorrected standard score | 106 (7) |
|  | Age-corrected standard score | 114 (8) |
|  | Fully-corrected T-score | 61 (6) |
| Picture vocabulary test age 3+ | Uncorrected standard score | 115 (12) |
|  | Age-corrected standard score | 111 (15) |
|  | Fully-corrected T-score | 58 (11) |
| Flanker inhibitory control and attention test age 12+ | Uncorrected standard score | 90 (6) |
|  | Age-corrected standard score | 88 (4) |
|  | Fully-corrected T-score | 42 (4) |
| List sorting working memory test age 7+ | Uncorrected standard score | 99 (9) |
|  | Age-corrected standard score | 108 (10) |
|  | Fully-corrected T-score | 54 (8) |
| Dimensional change card sort test age 12+ | Uncorrected standard score | 102 (5) |
|  | Age-corrected standard score | 108 (9) |
|  | Fully-corrected T-score | 54 (5) |
| Pattern comparison processing speed test age 7+ | Uncorrected standard score | 87 (13) |
|  | Age-corrected standard score | 95 (14) |
|  | Fully-corrected T-score | 45 (10) |
| Picture sequence memory test age 8+ | Uncorrected standard score | 102 (7) |
|  | Age-corrected standard score | 108 (5) |
|  | Fully-corrected T-score | 57 (2) |
| Oral reading recognition test age 8+ | Uncorrected standard score | 120 (9) |
|  | Age-corrected standard score | 130 (17) |
|  | Fully-corrected T-score | 73 (12) |

**eTable 11:** Generalized Anxiety Disorder (GAD-7), Post-traumatic stress disorder checklist (PCL-5), Patient Health Questionnaire (PHQ-15), Somatic Symptom Disorder (SSD-12) scores amongst survivors.

| **GAD-7 (n=5)** |  |
| --- | --- |
| Median score [IQI] | 0 [0-4] |
| No anxiety*^1^* | 3 |
| Mild anxiety*^1^* | 1 |
| Moderate anxiety*^1^* | 1 |
| **PCL-5 (n=5)** |  |
| Median score [IQI] | 2 [1-5] |
| Severe PTSD*^1^* | 1 |
| **PHQ-15 (n=5)** |  |
| Median score [IQI] | 4 [4-5] |
| Minimal Symptom Severity*^1^* | 3 (60) |
| Mild Symptom Severity*^1^* | 1 (20) |
| Medium Symptom Severity*^1^* | 0 |
| High Symptom Severity*^1^* | 1 (20) |
| **SSD-12 (n=5)** |  |
| Median score [IQI] | 7 [0-10] |
| Above cutoff for SSD*^1^* | 1 (20) |
| IQI = interquartile interval | |

**eTable 12:** Tobacco, Alcohol, Prescription Medication & other Substance use Tool (TAPS) part 1 amongst survivors who completed the tool (n=6). Answers are individual responses.

| In the past 12 months… | Daily or almost daily | Weekly | Monthly | Less Than Monthly | Never |
| --- | --- | --- | --- | --- | --- |
| 1. how often have you used any tobacco product (for example, cigarettes, ecigarettes, cigars, pipes, or smokeless tobacco)? |  |  |  |  | 6 |
| 2. how often have you had 4 or more drinks containing alcohol in one day? One standard drink is about 1 small glass of wine (5 oz), 1 beer (12 oz), or 1 single shot of liquor. (Note: This question should only be answered by males). |  |  | 1 | 2 | 3 |
| 3. how often have you used any drugs including marijuana, cocaine or crack, heroin, methamphetamine (crystal meth), hallucinogens, ecstasy/MDMA? |  |  | 1 | 1 | 4 |
| 4. how often have you used any prescription medications just for the feeling, more than prescribed or that were not prescribed for you? Prescription medications that may be used this way include: Opiate pain relievers (for example, OxyContin, Vicodin, Percocet, Methadone) Medications for anxiety or sleeping (for example, Xanax, Ativan, Klonopin) Medications for ADHD (for example, Adderall or Ritalin) |  |  |  |  | 6 |

**eTable 13:** Tobacco, Alcohol, Prescription Medication & other Substance use Tool (TAPS) part 2 amongst survivors who completed the tool (n=6). Answers are individual responses.

| In the past 3 months… | Number who answer ‘Yes’ out of 6 respondents |
| --- | --- |
| 1. Did you smoke a cigarette containing tobacco or use any other nicotine delivery product | 0 |
| 2. Consumption of drinks containing alcohol within the last 3 months | 3 |
| 3. Consuming 5 or more drinks containing alcohol in one day*^2^* | 1 |
| 4. Tried and failed to control, cut down or stop drinking in the past 3 months | 0 |
| 5. Someone has expressed concerns about your drinking in the last 3 months | 0 |
| 6. Use of marijuana (hash, weed) in the past 3 months | 2 |
| 7. Had a strong desire or urge to use marijuana at least once a week or more often in the past 3 months*^4^* | 0 |
| 8. Someone has expressed concern about your use of marijuana in the past 3 months*^4^* | 0 |
| 9. Cocaine, crack, or methamphetamine (crystal meth) use in the past 3 months | 0 |
| 10. Heroin use in the past 3 months | 0 |
| 11. Prescription medication use just for the feeling, more than prescribed or that were not prescribed for patient within the last 12 months | 0 |
| 12. Use of a prescription opiate pain reliever not as prescribed or that was not prescribed for you in the past 3 months | 0 |
| 13. Use of medication for anxiety or sleep not as prescribed or that was not prescribed for you within the past 3 months | 0 |
| 14. Use of medication for ADHD not as prescribed or that was not prescribed for you within the past 3 months | 0 |
| 15. Use of any other illegal or recreational drug in the past 3 months | 0 |

**eTable 14:** Charlson Comorbidity Index amongst 9 respondents overall, and by respondent type

| **CCI** | **Overall**, N = 9*^1^* | **Participant**, N = 6*^1^* | **SDM/ Other**, N = 3*^1^* |
| --- | --- | --- | --- |
| **Myocardial infarction (heart attack)** |  |  |  |
| No | 3 | 2 | 1 |
| Yes | 6 | 4 | 2 |
| **Heart failure (requiring treatment/ hospitalization)** |  |  |  |
| No | 5 | 3 | 2 |
| Yes | 4 | 3 | 1 |
| **Peripheral vascular disease** |  |  |  |
| No | 8 | 6 | 2 |
| Yes | 1 | 0 | 1 |
| **CVA or transient ischemic disease** |  |  |  |
| No | 9 | 6 | 3 |
| **Hemiplegia** |  |  |  |
| No | 9 | 6 | 3 |
| **Asthma, chronic lung disease, chronic bronchitis or emphysema** |  |  |  |
| No | 8 | 6 | 2 |
| Yes | 1 | 0 | 1 |
| **Diabetes (requires treatment)** |  |  |  |
| No | 6 | 4 | 2 |
| Yes | 3 | 2 | 1 |
| **End organ damage (from diabetes)** |  |  |  |
| No | 2 | 2 | 0 |
| Yes | 1 | 0 | 1 |
| **Moderate or severe renal disease** |  |  |  |
| No | 9 | 6 | 3 |
| **Chronic liver disease** |  |  |  |
| No | 9 | 6 | 3 |
| **Gastric or peptic ulcers** |  |  |  |
| No | 9 | 6 | 3 |
| **Cancer (other than basal cell skin cancer)** |  |  |  |
| No | 7 | 5 | 2 |
| Yes | 2 | 1 | 1 |
| **Metastatic tumor** |  |  |  |
| No | 9 | 6 | 3 |
| **Alzheimer’s, dementia from any etiology or any serious cognitive impairment** |  |  |  |
| No | 8 | 5 | 3 |
| Yes | 1 | 1 | 0 |
| **Rheumatic or connective tissue disease** |  |  |  |
| No | 8 | 5 | 3 |
| Yes | 1 | 1 | 0 |
| **HIV or AIDS** |  |  |  |
| No | 9 | 6 | 3 |
| **Hypertension** |  |  |  |
| No | 7 | 5 | 2 |
| Yes | 2 | 1 | 1 |
| **Decubitus ulcers, peripheral skin ulcers or repeated episodes of cellulitis** |  |  |  |
| No | 9 | 6 | 3 |
| **Depression** |  |  |  |
| No | 8 | 6 | 2 |
| Yes | 1 | 0 | 1 |
| **On warfarin, coumadin or any other blood thinners** |  |  |  |
| No | 5 | 2 | 3 |
| Yes | 4 | 4 | 0 |
| **Blood thinners other than warfarin and coumadin** |  |  |  |
| N/A | 6 | 3 | 3 |
| Apixaban | 2 | 2 | 0 |
| apo-apixaban | 1 | 1 | 0 |
| **CCI total score (mean (SD))** | **3 (3)** | **3 (3)** | **3 (4)** |

**eTable 15:** COVID Questionnaire administered at 6-month follow-up

| **COVID Questionnaire** | **n = 6** |
| --- | --- |
| **COVID Infection** |  |
| None | 4 |
| Once, confirmed by a physician/ diagnostic test other than PCR or antigen test | 2 |
| **COVID Symptoms (N= 2)** |  |
| Fever | 0 |
| Chest congestion, chest tightness, cough, or trouble breathing | 0 |
| Body aches and pains | 1 |
| Chills or shivering | 0 |
| Sore or painful throat, or congested, stuffy, runny, or dripping nose | 1 |
| Diarrhea | 0 |
| Constipation | 0 |
| Nausea | 0 |
| Abdominal pain | 0 |
| Feeling weak or more tired easily | 1 |
| Loss of smell | 0 |
| Loss of taste | 0 |
| Headaches | 0 |
| Cognitive problems | 0 |
| Memory fog | 0 |
| Other | 0 |
| **Currently still experiencing COVID symptoms (N=2)** |  |
| No | 2 |
| **Vaccinated against COVID** |  |
| Yes | 6 |
| **Number of COVID vaccines received** |  |
| 2 | 1 |
| 3 | 2 |
| 4 | 2 |
| 5 | 1 |

**eFigure 1:** Cleveland dot plot of the completeness of in-hospital data acquisition

**eFigure 2:** Serum levels of A) GFAP B) tau C) NfL and D) UCH-L1 were quantified on day 1, 2, 3, and 7 post cardiac arrest and at 6-month follow-up in eligible participants. Serial samples from individual patients are linked, with closed squares representing the group median levels per timepoint.

**eFigure 3**: Health-related quality of life as assessed by EQ-5D-5L (n=5)

.

**eFigure 4:** Stacked bar chart of the Patient Health Questionnaire (PHQ-9) (n=5)
